# Supplementary figures and images for: Analyses of Early ZIKV Genomes Are Consistent with Viral Spread from Northeast Brazil to the Americas
Source: Viruses. 2023 May 25;15(6):1236. doi: 10.3390/v15061236 (PMC10301521; doi:10.3390/v15061236)

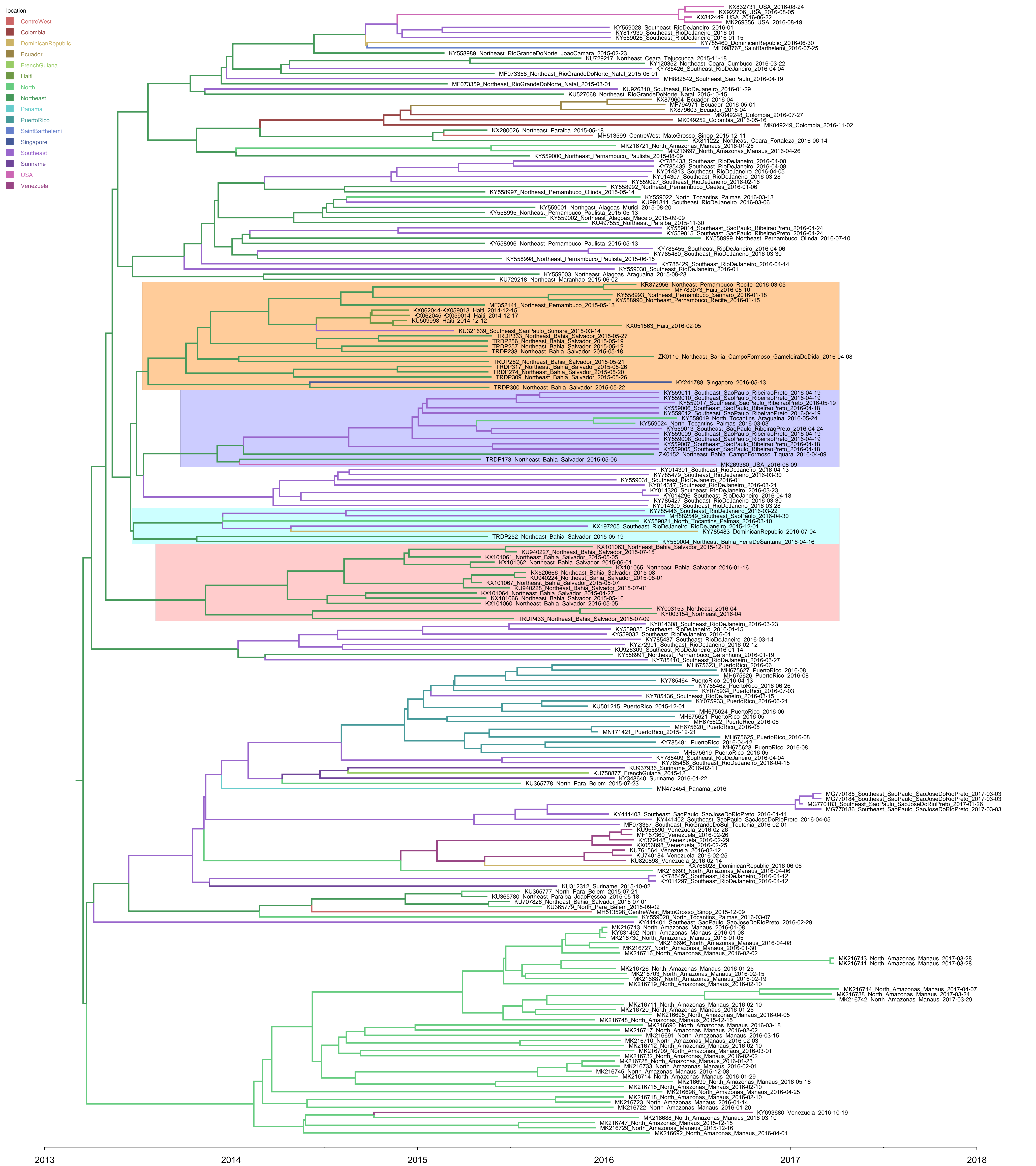

Supplement: Supplementary file 1 [file viruses-15-01236-s001.zip › ZIKVLaise_FigureS1_20230516LM.pdf]
